# Feasibility, Usability, and Preliminary Effectiveness of an mHealth App to Promote Screening Behaviors Among High-Risk Populations for Breast Cancer: Randomized Controlled Pilot Study

**DOI:** 10.2196/86429

**Published:** 2026-07-14

**Authors:** Linping Zhang, Mengjiao Xu, Xian Zhang, Xiaoxu Li, Shuo Wang, Fang Zhou, Jing Han

**Affiliations:** 1 School of Nursing, Xuzhou Medical University, No 209, Tongshan Road, Huangshan Street, Yunlong District, Xuzhou, China, 1 15896421496; 2 Department of Nursing, Affiliated Hospital of Xuzhou Medical University, Xuzhou, China

**Keywords:** breast cancer, cancer screening, high-risk populations, mobile health app, feasibility, usability

## Abstract

**Background:**

Breast cancer is a major public health challenge worldwide. Women at high risk for breast cancer are more likely to develop the disease; yet, screening participation remains. Mobile health interventions may improve breast health awareness and screening behaviors, but evidence in high-risk populations for breast cancer remains limited.

**Objective:**

This study aimed to evaluate the feasibility, usability, and preliminary signals of potential effectiveness of the “Ruaikang” mobile health app, designed to support breast health awareness and engagement with early screening among high-risk populations for breast cancer.

**Methods:**

In this pilot randomized controlled trial, eligible high-risk populations for breast cancer were randomly assigned (1:1) to a 4-week intervention using the “Ruaikang” app or a control group receiving standard breast cancer screening guidelines. Feasibility was assessed via recruitment and completion rates. Usability was measured using the System Usability Scale. Use data were collected through the app’s back-end system. Preliminary effectiveness was assessed through changes in willingness to participate in breast cancer screening, screening knowledge, health beliefs, and self-efficacy at both the fourth (T1) and eighth (T2) weeks. Semistructured interviews were conducted with a purposive subsample of intervention participants to explore user experience and suggestions for improvement.

**Results:**

Of 62 eligible women approached, 50 enrolled (recruitment rate 80.6%) and were randomized to intervention (n=25) and control (n=25) groups. In total, 19 participants in the intervention group completed the study (completion rate 76%), reflecting good feasibility. The mean usability score was 88.82 (SD 10.29), indicating high usability. Back-end use data showed that participants actively engaged during the intervention period. At T1, the intervention group showed preliminary signals of improvement in willingness to participate in breast cancer screening, screening knowledge, health beliefs, and self-efficacy, although these changes were less pronounced at T2. Qualitative interviews with 8 participants identified 2 core themes: “perceived value and acceptability of the app” and “system limitations and user-centered improvement needs.” Participants highlighted that the app was easy to use and content-rich and provided actionable feedback for optimizing interactive features, navigation, and content depth.

**Conclusions:**

The “Ruaikang” mobile health app demonstrated good feasibility and usability and provided preliminary signals of potential effectiveness in improving willingness to participate in breast cancer screening, screening knowledge, health beliefs, and self-efficacy among high-risk populations for breast cancer. Participants’ feedback indicates that the app aligns with users’ core needs and supports engagement with breast health information. Future work should refine the app based on user feedback and conduct larger-scale, longer-term randomized controlled trials to confirm and further validate the preliminary effectiveness observed.

## Introduction

Breast cancer presents a significant public health challenge for women globally. According to the International Agency for Research on Cancer [1], there were approximately 2.3 million new breast cancer cases and 670,000 deaths worldwide in 2022, making it the leading cause of cancer death among women after lung cancer. In China, approximately 350,000 new cases and 75,000 deaths were estimated in 2022, ranking it as the second leading cause of cancer-related mortality among Chinese women [2]. Cancer screening is an effective strategy for the early detection of breast cancer [3]. It involves the application of effective, simple, and economical breast examination to identify asymptomatic individuals at an early stage. As the most effective approach to enhancing early diagnosis rates, survival, and quality of life for patients, breast cancer screening is particularly vital for high-risk populations for breast cancer [4]. High-risk populations for breast cancer refer to women who carry established risk factors, such as a family history of breast cancer, relevant gene mutations, a history of benign breast disease, unhealthy lifestyle habits, smoking, and alcohol abuse [5]. Studies show that such individuals face a significantly elevated risk of developing breast cancer, which can be up to 10 times that of the general population [6].

Since the beginning of the 21st century, European Union countries such as the United Kingdom, Ireland, and Italy have actively promoted breast cancer screening programs, recommending that eligible women be screened every 2 to 3 years [7]. In 2009, the Chinese government launched a free “two cancers” (cervical cancer and breast cancer) screening program for eligible women and incorporated breast cancer screening into the national public health service in 2019 to encourage broader participation [8]. Despite these clear policy measures, participation in breast cancer screening remains low, particularly among high-risk populations for breast cancer. A study by Kırca et al [9] among first-degree relatives of patients with breast cancer in the largest hospital in southwestern Turkey revealed that fewer than 20% of participants had undergone screening. Similarly, a survey by Zhang et al [10] of 15,550 high-risk populations for breast cancer in Beijing showed that less than 50% were willing to participate in screening. Further findings from Zhao et al [11] highlighted low screening participation among first-degree relatives of patients with breast cancer, with only 6.7% of respondents performing breast self-examination once a month and 22.8% undergoing breast ultrasound or mammography once a year. According to the National Comprehensive Cancer Network (NCCN) Clinical Practice Guidelines [12], individuals identified as being at high risk for breast cancer include those with a lifetime risk of 20% or greater based on validated risk assessment models, a history of chest radiation therapy or breast cancer, the presence of high-risk breast lesions, or a family history suggestive of hereditary breast cancer. For this population, NCCN recommends earlier and more intensive screening, including annual mammography and breast magnetic resonance imaging, as well as clinical breast examinations every 6 to 12 months. The Chinese breast cancer screening guidelines [3] provide definitions and recommendations for high-risk populations for breast cancer that align closely with those of the NCCN. Therefore, it is imperative to develop and implement targeted interventions for high-risk populations for breast cancer to improve breast cancer screening participation, thereby alleviating the public health burden.

Previous studies suggested that factors such as breast cancer awareness, health beliefs, social support, and self-efficacy play significant roles in influencing women’s screening behaviors [9]. Researchers have developed various interventions, such as health education, peer support, patient navigation services, and decision assistance tools, to improve participation in breast cancer screening, and these studies have shown a positive promotion effect on screening participation [13-16]. Despite the demonstrated benefits of existing interventions, their implementation often depends on face-to-face delivery or printed materials, which require substantial personnel, time, and financial resources, thereby limiting scalability, flexibility, and long-term sustainability [17,18]. These challenges are particularly pronounced for women with demanding schedules, limited mobility, or those living in geographically dispersed areas, who may face difficulties in accessing such support [19]. In addition, most interventions focus on a single behavioral determinant, whereas women’s breast cancer screening behaviors are influenced by multiple interacting psychosocial factors [20]. Consequently, there is a need to develop more comprehensive, multicomponent intervention strategies that leverage digital platforms to improve accessibility, deliver timely support, and promote sustained participation in breast cancer screening.

With the global popularization of the internet, mobile health has become an integral component of contemporary health care [21]. As a key medium of mobile health, mobile health apps have been widely applied in multiple fields, including health monitoring, disease prevention, and chronic disease management [22-24]. To date, a considerable number of studies have been devoted to developing mobile health apps to promote breast cancer screening. For instance, Yusuf et al [25] developed a health education app tailored for women in northeastern Malaysia. By integrating content on breast anatomy, breast cancer knowledge, support group information, screening reminders, and culturally adapted educational materials, the app successfully enhanced screening confidence and willingness among the target population. Similarly, Roh et al [26] developed an app to encourage breast cancer screening among Native American people. This app offers breast cancer education, information on screening clinics, and regular text-message reminders and has shown promising results in real-world applications. However, existing apps primarily target the general population of healthy women, with limited attention to those high-risk populations for breast cancer. Compared to the general population, high-risk populations for breast cancer require more comprehensive breast health knowledge and more proactive health behaviors.

The research team developed a mobile health app named “Ruaikang” to provide breast cancer screening support resources for high-risk populations for breast cancer, enhance their breast health awareness, and promote participation in early screening. This pilot randomized controlled study aimed to evaluate the feasibility and usability of the “Ruaikang” mobile health app among high-risk populations for breast cancer and to explore its potential impact on promoting breast cancer screening behaviors.

## Methods

### Study Design

This pilot randomized controlled study was conducted from January to October 2025. The trial was registered in the Chinese Clinical Trial Registry (registration: ChiCTR2500108593) and reported in accordance with the CONSORT (Consolidated Standards of Reporting Trials) extension for pilot and feasibility trials (Checklist 1) [27].

### Participants and Setting

Participants were from a grade A tertiary hospital in eastern China. Convenience sampling was adopted to recruit the high-risk populations for breast cancer who visited the hospital during the study period. The inclusion criteria for the participants were as follows: (1) meeting the definition of high-risk populations for breast cancer per Chinese women’s breast cancer screening guidelines [3], including those with breast cancer susceptibility gene, family history of breast cancer (parents, children, and siblings), personal history of breast cancer, history of chest radiotherapy, and diagnosed with lobular carcinoma in situ, atypical ductal hyperplasia, or atypical lobular hyperplasia before 40 years; (2) women; (3) age ≥18 years; (4) possession of an Android smartphone and proficiency in its operation; and (5) those who voluntarily participated in this study and signed an informed consent form. The exclusion criteria were as follows: (1) physician-diagnosed confusion or cognitive impairment and (2) previous history of mental illness.

### Sample Size

In pilot studies, a minimum of 12 participants per group has been recommended to provide reasonable estimates of key parameters, such as the mean and variance, for planning future definitive trials [28]. In accordance with this guideline, the quantitative component of this study established a minimum sample size of 12 participants in each group. To account for a potential dropout rate of 20%, the final sample size was adjusted to at least 15 participants per group. For the qualitative component, participants were purposively sampled from the intervention group based on age, educational level, and app use patterns to ensure diverse and information-rich perspectives. The sample size was guided by the concept of information power [29], and data collection continued until sufficient information power was achieved to adequately address the study objectives.

### Procedures

The research team comprised 3 research assistants, 1 breast cancer care specialist, and 1 software engineer. The research assistants were primarily responsible for participant recruitment, intervention implementation, and data collection. The breast cancer care specialist provided clinical expertise and addressed participants’ medical questions. The software engineer was responsible for app maintenance and ensuring stable technical operation.

Research assistants screened potential participants in hospital wards and outpatient departments against the inclusion criteria. Eligible individuals received an explanation of the study’s background, purpose, and procedures. After providing informed consent, participants completed the baseline (T0) questionnaire and were randomly assigned to either the intervention group or the control group. The intervention group received a 4-week intervention via a mobile health app named “Ruaikang,” while the control group received the printed “Breast Cancer Screening Guidelines” manual [3]. Participants in the intervention group installed and registered the “Ruaikang” with the research assistants’ guidance. The assistants helped them log in, complete their personal profiles, and demonstrate the functionality of each module. Follow-up assessments were conducted with all participants at the fourth (T1) and eighth (T2) weeks. At T1, participants in the intervention group were also invited to participate in a semistructured interview about user experience and improvement suggestions for the app.

### Randomization and Blinding

A computer-generated random sequence was created by an independent statistician, who was not involved in participant recruitment or intervention delivery. Eligible participants were allocated in a 1:1 ratio to the intervention or control group. To ensure allocation concealment, the statistician prepared sequentially numbered, opaque, sealed envelopes, which were stored and managed by a research coordinator independent of participant enrollment and outcome assessment. Envelopes were opened sequentially only after participant enrollment and baseline data collection. Participants could not be blinded due to the nature of the intervention. Outcome assessors and research staff involved in follow-up and data collection remained blinded, and group assignment was not disclosed to assessors during follow-up to minimize detection bias.

### Intervention

**Figure 1.**
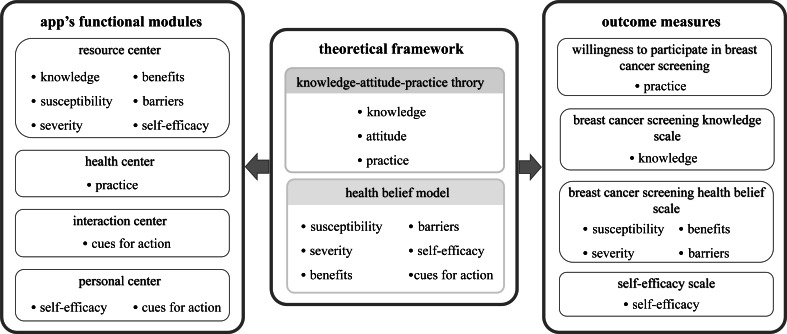
Conceptual framework.

The “Ruaikang” mobile health app was developed based on the knowledge-attitude-practice theory [30] and the health belief model (HBM) [31]. The knowledge-attitude-practice model outlines 3 stages for behavioral change: knowledge acquisition, attitude formation, and practice adoption. Knowledge enables attitude shifts, which drive behavioral change for health goals. HBM explains health behaviors through 6 belief dimensions: perceived susceptibility, perceived severity, perceived benefits, perceived barriers, self-efficacy, and cues for action. In this study, “knowledge” refers to understanding of breast cancer pathology and early screening protocols. “Attitude” refers to the cultivation of positive beliefs and attitudes toward early screening participation among high-risk populations for breast cancer, with conceptual alignment to core HBM dimensions. “Practice” refers to adoption of regular breast cancer screening behaviors by high-risk populations for breast cancer, informed by acquired knowledge and reinforced attitudes. This theoretical framework informed both the design of the app’s functional modules and the selection of outcome measures, as illustrated in Figure 1. The app comprises 2 core systems: the user side and the back-end management system. The user side is designed to deliver breast health education, early screening guidance, health behavior promotion, and psychosocial support for high-risk populations for breast cancer, including 4 core functional modules: the resource center, health center, interaction center, and personal center. The back-end management system is designed to oversee and control the overall operation of the app. The detailed information on the app’s development process, user side features, and back-end management functions is shown in Multimedia Appendix 1.

### Outcome Measures

#### The Feasibility and Usability of the App

The feasibility and usability of the app are as follows:

- Feasibility: The feasibility of the app was assessed using 2 key indicators: recruitment rate and completion rate. The recruitment rate was defined as the percentage of volunteers who participated in the study among all individuals who met the eligibility criteria. The completion rate referred to the percentage of participants who finished the entire intervention out of all those who agreed to participate. Based on previous studies [32], the feasibility threshold was set as follows: recruitment rate ≥80% and completion rate ≥75%.
- Usability: The usability of the app was assessed using the System Usability Scale [33]. The scale comprises 10 items; responses were scored on a 5-point Likert scale, and each item yielded a value between 0 and 4. The sum of all item scores is multiplied by 2.5 to produce a total score ranging from 0 to 100. If the total score is ≥70 [34], the usability of the app is considered to meet the standard. The Cronbach α value of the scale was 0.84, and in this study, it was 0.87.
- Use: Researchers collected users’ use duration, the number of resource page visits, comments posted, and health consultations through the app’s back-end management system.

#### The Preliminary Effectiveness of the App

##### Primary Outcome

Willingness to participate in breast cancer screening was assessed using items embedded within the general information questionnaire.

##### Secondary Outcomes

###### Breast Cancer Screening Knowledge

The Breast Cancer Screening Knowledge Scale [35] was used to assess participants’ knowledge of breast cancer and screening. This instrument was developed based on the Chinese breast cancer screening guidelines and authoritative educational materials and has been publicly used in China [36,37]. This questionnaire comprises 17 items assessing knowledge across 3 domains: high-risk factors for breast cancer, clinical manifestations, and early screening methods. All items are presented as true or false statements. Each correct response earns 1 point, with no points awarded for incorrect answers. The total score ranges from 0 to 17 points, with higher scores indicating a higher level of knowledge about breast cancer and screening. The Cronbach α value of the scale was 0.72 [35], and the Cronbach α in this study was 0.82.

###### Breast Cancer Screening Health Belief

The Breast Cancer Screening Health Belief Scale [38], which was developed based on the HBM framework, was used to assess women’s beliefs and attitudes toward breast cancer screening. The scale consists of 12 items, each scored on a 5-point Likert scale (ranging from 1 to 5). The scale comprised 4 dimensions: perceived susceptibility, perceived severity, perceived benefits, and perceived barriers. Total scores for each dimension ranged from 2 to 10 for perceived susceptibility, 3 to 15 for both perceived severity and perceived benefits, and 4 to 20 for perceived barriers. Higher scores indicated stronger health beliefs regarding breast cancer screening. The scale has been publicly used [39]. The Cronbach α value of the scale was 0.89 [38], and in this study, it was 0.70.

###### Self-Efficacy

The General Self-Efficacy Scale [40] was used to assess women’s confidence in their ability to complete breast cancer screening when faced with various challenges. The General Self-Efficacy Scale has been applied in patients with cancer and has demonstrated good reliability and validity [41]. The scale was translated and culturally adapted into Chinese by Wang [42]. It consists of 10 items, and responses to the questions were scored on a 4-point Likert scale. The total score ranges from 10 to 40 points, with higher scores indicating higher levels of self-efficacy. The Cronbach α value of the scale was 0.87, and in this study, it was 0.95.

### User Experience and Improvement Suggestions for the App

The interview guide was developed based on the unified theory of acceptance and use of technology framework [43], prior usability and user experience studies of mobile health apps [44], and the specific objectives of this study. It was further refined through discussion among the research team and consultation with oncology and mobile health experts to ensure that the questions were clear, relevant, and aligned with the study goals. The interview guide included the following questions: What is your overall experience with the app? What benefits did you gain from using the app? Which aspects of the app do you believe need improvement? Would you recommend the app to others? Why?

### Data Collection

After receiving standardized training, the research team members conducted data collection following unified instructions. Participants in both groups completed questionnaires at T0, T1, and T2, with each questionnaire taking approximately 10‐15 minutes to complete. Data at T0 were collected through face-to-face paper questionnaires, while data at T1 and T2 were collected via telephone, electronic questionnaires, or face-to-face administration. After retrieving the questionnaires, researchers promptly checked the completeness and validity of the responses and conducted supplementary investigations when necessary. All collected data were anonymized, securely stored, and used solely for the purposes of this study. The research process strictly adhered to ethical principles, ensuring participants’ voluntariness, confidentiality, and data security, while minimizing potential risks.

Feedback regarding the experience and improvement suggestions for the app were collected from participants in the intervention group through face-to-face or telephone interviews. The interviews were conducted by 2 researchers (LZ and MX) with 3 years of clinical experience in oncology and training in qualitative research. The data collection process was as follows: (1) the purpose and significance of the interview were explained to participants who had completed the intervention, written informed consent was obtained, and permission to record the interview was granted; and (2) the researchers conducted one-to-one, semistructured interviews while audio-recording each session. Each interview lasted approximately 20‐30 minutes.

### Data Analysis

Quantitative data were analyzed using SPSS software (version 27; IBM Corp). Descriptive statistics were used to summarize participants’ sociodemographic characteristics, as well as the feasibility, usability, and use of the app, and the outcome variables of effectiveness at different time points. Continuous variables were presented as mean and SD, while categorical variables were presented as frequency and percentage. Differences in baseline characteristics between groups were examined using 2-tailed independent-samples *t* tests and chi-square statistics. In accordance with the intention-to-treat principle, generalized estimating equations (GEE) were used to compare changes in outcome variables between the control and intervention groups across time points. In the GEE analysis, missing data were handled using multiple imputation, and a sensitivity analysis was conducted to assess the robustness of results by comparing outcomes before and after handling missing data. The level of statistical significance was set at *P*<.05.

Qualitative data analysis was initiated following the first interview. Upon completion of each session, the interviewing researchers reviewed the audio recordings, performed verbatim transcription, and imported the transcripts into NVivo (version 12.0; QSR International) within 24 hours. Analysis was conducted following a coding-reliability thematic analysis approach to ensure transparency, objectivity, and reproducibility of the findings [45]. The research team repeatedly read all transcripts to become thoroughly familiar with the data and held meetings to discuss preliminary impressions, establishing a shared understanding to guide systematic coding. Based on the research questions, a structured initial codebook was developed, with each code assigned a clear name, operational definition, and inclusion or exclusion criteria. Two researchers (LZ and MX) independently applied this codebook to a randomly selected subset of transcripts for pilot coding. Intercoder reliability was assessed using both percentage agreement and Cohen κ (initial agreement=85%; κ=0.78). Discrepancies were discussed and resolved by referring to the original transcripts, and the codebook was revised and finalized for use in coding the full dataset. The finalized codebook was then applied independently to the remaining transcripts. Any novel or marginal data encountered during coding were documented for periodic review but did not modify the established coding framework. Once coding was complete, all coded data were exported, and the researchers examined the codes in relation to their frequency, patterns of co-occurring codes, and coverage within the dataset to identify potential themes. Each theme was then reviewed against the original transcripts to ensure that it accurately represented the data and was supported by sufficient evidence. Final themes were refined and clearly defined, with descriptive labels that accurately reflected the content of the data while avoiding overinterpretation. These descriptive themes directly addressed the research questions and provided reliable evidence. Data collection was concluded once the researchers determined that sufficient information power had been achieved. Additionally, both researchers selected representative quotations from the dataset and translated them into English. In cases of ambiguity during translation, the team consulted professional translation experts to ensure semantic accuracy and preserve the original intent of the participants’ statements.

### Ethical Considerations

This study received approval from the ethics committee of Xuzhou Mining Group General Hospital (approval 2025082501). All procedures performed were in accordance with the ethical principles of the Declaration of Helsinki. Written informed consent was obtained from all participants before randomization. Prior to the study, the research objectives and procedures were thoroughly explained to participants, and all inquiries were addressed. Participants were assured of the strict confidentiality of their personal information and were informed of their right to withdraw unconditionally at any time. As compensation for their time and contribution, each participant received a gift worth US $7.35 upon completion of data collection.

## Results

### Recruitment and Characteristics of the Participants

**Figure 2.**
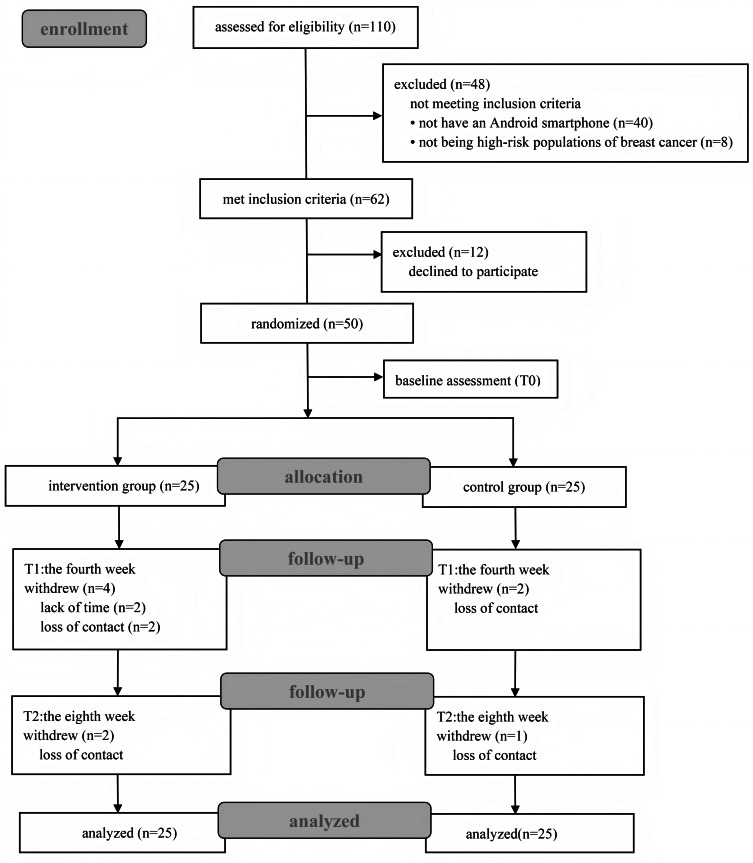
CONSORT flow diagram. CONSORT: Consolidated Standards of Reporting Trials.

A total of 110 women were assessed for eligibility, with 62 meeting the inclusion criteria and 48 excluded due to not having an Android smartphone (n=40) or not being high-risk populations for breast cancer (n=8). Among the eligible participants, 12 declined to participate, resulting in 50 participants who were successfully enrolled and randomly assigned to either the intervention or control group. During follow-up, 6 participants in the intervention group withdrew due to lack of time or loss of contact, while 3 participants in the control group withdrew due to loss of contact. The recruitment flow and reasons for dropout are detailed in Figure 2. Baseline characteristics of participants in both groups are shown in Table 1, with no significant differences across measures, indicating comparability between groups.

**Table 1.** Baseline characteristics of the participants (N=50).

Variables	Intervention group (n=25)	Control group (n=25)	Statistics	*P* value
Age (years), mean (SD)	43.48 (8.90)	41.20 (8.97)	*t*_48_=−0.90	.37
Ethnicity, n (%)			*χ*^2^_1_=0.2	>.99
Han	23 (92)	22 (88)		
Other	2 (8)	3 (12)		
Educational attainment, n (%)			*χ*^2^_2_=1.8	.44
Junior high school and below	6 (24)	10 (40)		
High school or junior college	12 (48)	8 (32)		
Bachelor's degree and above	7 (28)	7 (28)		
Employment status, n (%)			*χ*^2^_2_=0.6	.84
Employed	16 (64)	17 (68)		
Freelancer	4 (16)	5 (20)		
Retired	5 (20)	3 (12)		
Marital status, n (%)			*χ*^2^_1_=0.4	>.99
Married	24 (96)	23 (92)		
Unmarried	1 (4)	2 (8)		
Medical insurance, n (%)			*χ*^2^_1_=0.0	>.99
Yes	24 (96)	24 (96)		
No	1 (4)	1 (4)		
Family history of breast cancer, n (%)			*χ*^2^_1_=0.4	.54
Yes	16 (64)	18 (72)		
No	9 (36)	7 (28)		
Personal history of breast cancer, n (%)			*χ*^2^_1_=0.6	.44
Yes	3 (12)	5 (20)		
No	22 (88)	20 (80)		
Diagnosed with LCIS^a^, ADH^b^, or ALH^c^ before 40 years, n (%)			*χ*^2^_1_=0.9	.33
Yes	8 (32)	5 (20)		
No	17 (68)	20 (80)		
History of breast cancer screening, n (%)			*χ*^2^_1_=0.1	>.99
Yes	7 (28)	6 (24)		
No	18 (72)	19 (76)		
Willingness to participate in breast cancer screening, n (%)			*χ*^2^_1_=0.8	>.99
Yes	13 (52)	12 (48)		
No	12 (48)	13 (52)		
Breast cancer screening knowledge, mean (SD)	8.12 (2.80)	8.48 (2.55)	*t*_48_=0.48	.64
Perceived susceptibility, mean (SD)	6.20 (0.91)	6.72 (1.06)	*t*_48_=1.86	.07
Perceived severity, mean (SD)	7.40 (1.85)	8.12 (1.56)	*t*_48_=1.49	.14
Perceived benefits, mean (SD)	13.04 (1.17)	12.72 (1.79)	*t*_48_=−0.75	.46
Perceived barriers, mean (SD)	13.64 (2.43)	12.52 (2.37)	*t*_48_=−1.65	.11
Self-efficacy, mean (SD)	22.36 (5.25)	23.24 (5.39)	*t*_48_=0.59	.56

a LCIS: lobular carcinoma in situ.

b ADH: atypical ductal hyperplasia.

c ALH: atypical lobular hyperplasia.

### Feasibility and Usability of the App

The feasibility of the app was illustrated by the CONSORT flowchart (Figure 2). The recruitment rate was 80.6% (50/62), and the completion rate was 76% (19/25).

The average total score on the System Usability Scale was 88.82 (SD 10.29), and the score of each item is shown in Table 2. Among the odd-numbered items on the scale, the highest score was for the item “I think that I would like to use this system frequently” (mean 3.89, SD 0.32), indicating the strongest agreement among participants with this statement. Among the even-numbered items, the lowest score was for the item “I think that I would need the support of a technical person to be able to use this system” (mean 3.05, SD 1.03), reflecting the highest level of disagreement among participants.

**Table 2.** The average score of the System Usability Scale (n=19).

Items	Mean (SD)
I thought that I would like to use this system frequently	3.89 (0.32)
I found the system unnecessarily complex	3.58 (0.61)
I thought the system was easy to use	3.74 (0.45)
I thought that I would need the support of a technical person to be able to use this system	3.05 (1.03)
I found the various functions in this system were well integrated	3.74 (0.45)
I thought there was too much inconsistency in this system	3.42 (0.51)
I would imagine that most people would learn to use this system very quickly	3.68 (0.58)
I found the system very cumbersome to use	3.47 (0.51)
I felt very confident using the system	3.74 (0.45)
I needed to learn a lot of things before I could get going with this system	3.21 (0.86)

During the intervention period, the app use of participants in the intervention group is shown in Table 3. The average app use duration was 2.03(SD 0.77) hours, the average number of resource page visits was 30.42 (SD 10.93), the average number of comments posted was 1.05 (SD 1.27), and the average number of health consultations was 0.89 (SD 0.99).

**Table 3.** The use of the app during the intervention period (n=19)^a^.

Participants^b^	Use duration (hours)	Resource page visits, n	Comments posted, n	Health consultations, n
S1	3	46	3	2
S2	2	30	2	1
S3	2.5	37	3	2
S4	3.5	48	3	2
S5	3	45	2	1
S6	1	31	0	0
S7	2	29	0	0
S8	2.5	38	1	3
S9	1	16	0	0
S10	1.5	23	0	0
S11	2	16	0	0
S12	2	30	1	1
S13	1.5	23	0	0
S14	1	16	0	0
S15	2.5	37	2	2
S16	2	30	0	1
S17	3	45	3	2
S18	1.5	23	0	0
S19	1	15	0	0

a Use duration: mean 2.03, SD 0.77; resource page visits: mean 30.42, SD 10.93; comments posted: mean 1.05, SD 1.27; health consultations: mean 0.89, SD 0.99.

b S1 to S19 refer to the participants who completed the study.

### Preliminary Signals of Effectiveness

As shown in Table 4, the proportion of participants willing to participate in breast cancer screening increased from T0 to T1 in both groups, with a more pronounced increase in the intervention group. However, this proportion declined in the intervention group from T1 to T2. As shown in Table 5, GEE analysis indicated that at T1, participants in the intervention group tended to report higher willingness compared to those in the control group (odds ratio 4.889, 95% CI 1.343-17.801), while by T2, this difference was less pronounced (odds ratio 2.667, 95% CI 0.717-9.912).

**Table 4.** Descriptive statistics for outcome measures at each time point for the intervention and control groups (number of each group=25).

Variables	T0^a^		T1^b^		T2^c^	
	Intervention group	Control group	Intervention group	Control group	Intervention group	Control group
Willingness to participate in breast cancer screening, n (%)						
Yes	13 (52)	12 (48)	22 (88)	15 (60)	20 (80)	15 (60)
No	12 (48)	13 (52)	3 (12)	10 (40)	5 (20)	10 (40)
Breast cancer screening knowledge, mean (SD)	8.12 (2.80)	8.48 (2.55)	16.45 (1.74)	9.25 (2.13)	15.20 (2.24)	9.75 (1.88)
Perceived susceptibility, mean (SD)	6.20 (0.91)	6.72 (1.06)	8.06 (1.09)	6.74 (1.00)	7.88 (1.04)	6.96 (0.84)
Perceived severity, mean (SD)	7.40 (1.85)	8.12 (1.56)	10.43 (1.19)	8.52 (1.20)	10.29 (1.49)	9.11 (1.23)
Perceived benefits, mean (SD)	13.04 (1.17)	12.72 (1.79)	14.76 (0.95)	13.20 (1.27)	14.46 (1.09)	13.30 (1.13)
Perceived barriers, mean (SD)	13.64 (2.43)	12.52 (2.37)	10.30 (1.60)	12.53 (2.07)	10.65 (1.80)	12.20 (1.41)
Self-efficacy, mean (SD)	22.36 (5.25)	23.24 (5.39)	33.79 (4.61)	23.45 (4.88)	31.21 (4.87)	23.89 (4.47)

a T0: baseline.

b T1: the fourth week.

c T2: the eighth week.

**Table 5.** Generalized estimating equation models for the comparison of outcomes across time between the 2 groups (number of each group=25).

Variables and parameters	Statistics	*P* value
Willingness to participate in breast cancer screening, OR^a^ (95% CI)		
Group	1.000 (0.330 to 3.033)	>.99
T1^b^	1.625 (0.966 to 2.735)	.07
T2^c^	1.625 (0.966 to 2.735)	.07
Group*T1	4.889 (1.343 to 17.801)	.02
Group*T2	2.667 (0.717 to 9.912)	.14
Breast cancer screening knowledge, β^d^ (95% CI)		
Group	−0.360 (−1.816 to 1.096)	.63
T1	0.773 (0.055 to 1.491)	.03
T2	1.268 (0.168 to 2.368)	.02
Group*T1	7.557 (6.031 to 9.083)	<.001
Group*T2	5.813 (4.055 to 7.570)	<.001
Perceived susceptibility, β (95% CI)		
Group	−0.520 (−1.058 to 0.018)	.06
T1	0.022 (−0.219 to 0.264)	.86
T2	0.238 (−0.011 to 0.487)	.06
Group*T1	1.835 (1.272 to 2.398)	<.001
Group*T2	1.440 (0.848 to 2.032)	<.001
Perceived severity, β (95% CI)		
Group	−0.720 (−1.650 to 0.210)	.13
T1	0.396 (−0.165 to 0.957)	.17
T2	0.995 (0.289 to 1.700)	.01
Group*T1	2.634 (1.721 to 3.547)	<.001
Group*T2	1.891 (0.713 to 3.070)	<.01
Perceived benefits, β (95% CI)		
Group	0.320 (−0.502 to 1.142)	.45
T1	0.479 (0.061 to 0.896)	.03
T2	0.576 (0.017 to 1.135)	.04
Group*T1	1.236 (0.581 to 1.891)	<.001
Group*T2	0.840 (0.055 to 1.626)	.04
Perceived barriers, β (95% CI)		
Group	1.120 (−0.182 to 2.422)	.09
T1	0.007 (−0.447 to 0.462)	.98
T2	−0.320 (−1.067 to 0.427)	.40
Group*T1	−3.351 (−4.502 to −2.199)	<.001
Group*T2	−2.670 (−4.007 to −1.262)	<.001
Self-efficacy, β (95% CI)		
Group	−0.880 (−3.769 to 2.009)	.55
T1	0.291 (−0.528 to 0.949)	.58
T2	0.647 (−1.006 to 2.299)	.44
Group*T1	11.224 (8.952 to 13.496)	<.001
Group*T2	8.206 (5.598 to 10.813)	<.001

a OR: odds ratio.

b T1: the fourth week.

c T2: the eighth week.

d β: regression coefficient.

Breast cancer screening knowledge scores increased from T0 to T1 in both groups, with a greater improvement observed in the intervention group. Although the intervention group’s scores declined from T1 to T2, they remained above the baseline (Table 4). As shown in Table 5, GEE analysis indicated that knowledge scores in the intervention group tended to be higher than those in the control group at T1 (β=7.557, 95% CI 6.031-9.083) and T2 (β=5.813, 95% CI 4.055-7.570).

The intervention group showed increases from T0 to T1 in scores for perceived susceptibility, severity, and benefits. Although a slight decline occurred by T2, scores remained above baseline. Conversely, perceived barriers decreased at T1 before a slight rebound at T2. Throughout the study, scores in the control group showed minimal fluctuation (Table 4). Analysis of GEE indicated that changes in health belief scores across all dimensions tended to be greater in the intervention group compared with the control group (Table 5).

Regarding self-efficacy, scores in the intervention group increased from T0 to T1. Although a slight decline was observed at T2, they remained above baseline. Changes in the intervention group tended to be greater than those in the control group, which showed smaller fluctuations (Table 4). According to GEE analysis (Table 5), the improvement in self-efficacy in the intervention group was consistently greater than in the control group at both T1 (β=11.224, 95% CI 8.952-13.496) and T2 (β=8.206, 95% CI 5.598-10.813). Given the pilot and formative nature of this study, these findings should be interpreted as exploratory trends rather than conclusive evidence of effectiveness.

To evaluate the robustness of the findings to missing data, a sensitivity analysis was conducted by comparing the GEE results from the multiple imputed dataset with those obtained from the original dataset. Overall, the direction of the intervention effects was largely consistent across analyses, indicating that the findings were stable regardless of the method used to handle missing data. The detailed results of the sensitivity analysis are presented in Multimedia Appendix 2.

### User Experience and Improvement Suggestions for the App

#### Overview

In total, 8 participants were interviewed. The participants ranged in age from 35 to 57 years (mean 45.38 years, SD 6.99), used the app for a total of 1 to 4 hours (mean 2.25 hours, SD 1.04), and interview duration ranged from 21 to 27 minutes (mean 23.75 minutes, SD 2.43). Detailed demographic and use characteristics are presented in Multimedia Appendix 3. Analysis of the interview transcripts initially generated 20 codes. Based on recurring patterns and relationships among the coded data, these codes were grouped into 4 subthemes, which were subsequently refined into 2 core themes: “perceived value and acceptability of the app” and “system limitations and user-centered improvement needs.” The details are shown in Multimedia Appendix 4.

#### Theme 1: Perceived Value and Acceptability of the App

Participants provided positive feedback on the “Ruaikang” mobile health app, with its strengths and value summarized into 2 subthemes: hybrid approach to promote effectiveness and long-term or sustained use and significant health promotion effectiveness.

##### Hybrid Approach to Promote Effectiveness and Long-Term or Sustained Use

Participants reported that the app’s content was professionally rigorous, clearly illustrated, and highly practical. Its information architecture was logically structured to cover the entire spectrum of breast health management, including disease awareness, prevention strategies, and daily self-management. They also reported that the app was simple and easy to use. The combination of high-quality content and user-friendly design enhanced participants’ engagement with the app and fostered a strong willingness to continue using it. Furthermore, participants expressed a willingness to actively recommend the app to others, citing its professional content, practical utility, and ongoing updates. They viewed it as a valuable tool for educating friends and family about breast health and reducing anxiety, which further reinforced its potential for sustained use and wider dissemination.

The application provides comprehensive content, from disease awareness and prevention strategies to daily self-management strategies, eliminating the need to consult disparate online sources. This integrated approach offers considerable convenience. The detailed self-examination video facilitates effective self-screening at home, promoting earlier detection of potential issues. Furthermore, the user experience is very comfortable, with no unnecessary clutter, making all features readily accessible.

[S4]

People are paying more attention to self-care these days, and knowing about breast health is important for protection. The application provides professional, readily accessible information. I plan to use it often if it stays updated ... It is comprehensive and practical, useful for helping my mother and friends gain knowledge and reduce anxiety. I’ll definitely recommend it.

[S2]

##### Significant Health Promotion Effectiveness

By providing professional content and practical features, the app contributed significantly to participants’ breast health knowledge and awareness, while also supporting the development of healthier lifestyle habits.

The application taught me the warning signs for breast cancer and how to prevent it. It clearly explained the screening process, including when and how to get checked, which was so valuable. Thanks to the self-examination guide, I now check myself every week. It makes me feel much more secure.

[S5]

Now I make more mindful, healthier choices, like buying more of the recommended vegetables when shopping for groceries, controlling my dinner portions, and sticking to my daily health tracking. It’s all helping me build solid habits.

[S8]

### Theme 2: System Limitations and User-Centered Improvement Needs

Participants identified limitations in the “Ruaikang” mobile health app pertaining to its functionality, content, and user interaction and proposed constructive optimizations. This theme can be summarized into 2 subthemes: lack of active engagement and interactions and functional and content-related limitations.

#### Lack of Active Engagement and Interactions

Participants identified several issues related to active engagement and interaction within the app. They proposed adding an AI-powered question-and-answer system or chatbot to facilitate timely communication and support. Participants also recommended incorporating a check-in incentive mechanism to enhance user engagement. In addition, they highlighted the absence of a platform for user-to-user communication and suggested creating a community forum to facilitate discussion and experience sharing.

I would like to see AI-powered features added, such as an instant Q&A system or a chatbot ... Additionally, incorporating incentives into the Health Center, for example, rewarding users with a free consultation upon completing a health plan, would further boost engagement and support self-management.

[S3]

The interactive center lacks a section for users to discuss issues, and users cannot view others’ shared experiences or respond to questions. I hope to see an interactive space where people can talk about problems they encounter and learn how others have solved them.

[S6]

#### Functional and Content-Related Limitations

Participants highlighted several limitations related to the app’s interface and content. They noted ambiguous section boundaries, difficulty locating desired information, a lack of direct access to frequently used content, and highly repetitive visual elements, which reduced usability and visual comfort. Participants recommended optimizing page layouts, improving content organization, providing quicker access to commonly used information, and enhancing design diversity to improve the user experience. Regarding content presentation, participants reported that locating key information within videos was time-consuming and suggested providing concise summaries, such as text descriptions, slide decks, or flowcharts, to facilitate information retrieval and comprehension. Participants also perceived limitations in the existing content, particularly within the health center, and expressed a need for more detailed explanations of specialized topics, such as breast cancer staging, symptoms, and policy-related information. In addition, they emphasized the importance of regularly updating content to ensure that information remains current and relevant.

The dietary recommendations are buried within a broad category and lack a direct access point. Creating a more visible shortcut for such frequently used content would save significant time ... It would be helpful to summarize the key points from the video into a separate format, like a flowchart or a slide deck, to distill the core content for quick comprehension.

[S5]

Regarding breast cancer staging, I hope to see clearer explanations for each stage’s definition, symptoms, and management approaches. The current symptom illustrations are only in cartoon form, I recommend adding realistic image options to make the content more comprehensive and meet diverse needs.

[S4].

## Discussion

### Principal Findings

“Ruaikang” as a pioneering mobile health app designed specifically to promote early screening behaviors among high-risk populations for breast cancer demonstrated good feasibility and usability in this study. Preliminary findings suggested trends toward improvements in participants’ willingness to participate in breast cancer screening, screening knowledge, health beliefs, and self-efficacy, although these results should be interpreted cautiously, given the exploratory, formative nature of the pilot study. Qualitative analysis further suggested that participants perceived its strengths in content expertise, user-friendly operation, practical functionality, and health promotion impact. However, it also identified areas for improvement in functional optimization, user interaction, and content presentation, which can inform subsequent refinement and development.

In this study, the recruitment rate reached 80.6% (50/62), and the completion rate was 76% (19/25), exceeding the feasibility thresholds for mobile health apps (recruitment rate ≥80% and completion rate ≥75%) [32], indicating that “Ruaikang” demonstrates strong feasibility among high-risk populations for breast cancer. High engagement levels not only confirm that the app’s core functions effectively align with the health needs of the target group [46] but also reflect the potential of mobile health tools in early breast cancer prevention [47]. Moreover, qualitative findings corroborate these results, as participants reported the app’s content to be practical and its features relevant to their needs. When conducting large-scale randomized controlled trials in the future, participation and completion rates can be further enhanced through optimizing recruitment strategies [32], establishing strategies to support sustained engagement [48], and others.

The usability score of “Ruaikang” was 88.82 (SD 10.29), which is similar to the score (83.20) reported for the mobile health app developed by Rezaee et al [49], indicating that “Ruaikang” demonstrates good usability. Participants reported that the app was easy to operate, intuitive, and usable without professional guidance. These findings were further supported by use data and qualitative feedback, which together indicated active engagement and ease of navigation, reflecting strong learnability and user-friendliness. Several factors may explain this high level of usability. First, “Ruaikang” was specifically developed for high-risk populations for breast cancer, allowing the content to closely align with their unique needs, concerns, and risk perceptions. Prior studies have emphasized that tailoring health information to a defined target group is a key determinant of usability and engagement, as users are more likely to interact with information they perceive as personally relevant [50]. Second, the structured and comprehensive design of the app integrates educational content, self-management guidance, and practical tools in a logical and accessible manner, reducing the need to seek information from multiple sources and thereby enhancing convenience. Systematic reviews of mobile health usability note that clear organization and efficient information flow are central to perceived usability, as they reduce cognitive load and support efficient task completion [51]. Finally, the inclusion of multimedia resources, such as videos and images, improves clarity and engagement, providing a more interactive and motivating learning experience than text-based apps. Empirical studies suggest that multimedia elements can enhance understanding and make complex health information easier to process, which in turn supports sustained interaction with mobile health tools [52]. These features likely contributed to participants’ strong engagement and willingness to use the app frequently, suggesting that tailored content, structured design, and interactive elements can significantly enhance the usability of mobile health interventions targeting high-risk populations for breast cancer. Nevertheless, the relatively high feasibility and usability observed in this study may be partly attributable to the recruitment of participants from a single tertiary hospital, all of whom owned and regularly used smartphones. Such participants likely possessed higher digital literacy and greater familiarity with mobile technologies, which facilitated their engagement with the app and led to more positive evaluations [53].

This study provides preliminary signals of improvement in willingness to participate in breast cancer screening, screening knowledge, health beliefs, and self-efficacy among high-risk populations for breast cancer, as supported by both quantitative and qualitative findings. These results are consistent with a systematic review of Salman et al [47], which evaluated mobile health apps in cancer screening and reported potential effects on screening participation and health outcomes. Importantly, several key outcome scores declined at the follow-up (T2) assessment, suggesting that the initial gains were not fully sustained. This pattern is consistent with prior research on mobile health interventions, which has shown that benefits often peak immediately after the intervention and may diminish over time without ongoing support, highlighting the potential importance of sustained engagement for maintaining behavior change [54]. In addition, the relatively wide CIs further limit the precision of these estimates and should be considered when interpreting the observed trends. Qualitative insights suggest that the decline in engagement and outcome scores at T2 may be interpreted as reflecting participants’ need for more interactive and dynamically updated content, as well as motivational elements. These findings may highlight the need for strategies that extend beyond short-term initiation to promote continued engagement and behavior reinforcement. Future iterations of the app could incorporate more interactive and updated content, gamification mechanisms, personalized reminders, and theory-driven features to strengthen long-term engagement and maintenance of screening-related behaviors. Importantly, the primary outcome of this study was willingness to participate in screening, which reflects behavioral intention rather than actual screening uptake. However, these findings should be interpreted as hypothesis-generating rather than confirmatory, given the pilot nature of the trial. Future studies with larger samples and longer follow-up periods using fully powered randomized controlled trial designs are warranted to confirm these findings.

In addition to recognizing the app’s strengths, participants also identified several areas for improvement, including functional and interaction features, interface and content presentation, and the depth and frequency of content updates. These comments do not negate that the app meets the core needs of high-risk populations for breast cancer and instead reflect users’ expectations for greater convenience, enhanced interactivity, and content relevance in real-world use. The low frequency of interactive feature use may be related to inconspicuous entry points, delayed feedback, and limited opportunities for peer interaction. Consistent with research on mobile health interventions, users prefer features that facilitate active engagement and meaningful interaction, which are critical for sustained use [44]. Navigation and content presentation issues suggest a misalignment between the app’s linear, chapter-based knowledge structure and the fragmented, problem-focused use patterns of high-risk populations for breast cancer. Research on user-centered design for breast cancer-related apps has highlighted that clear navigation tools and well-organized content are key factors influencing usability and engagement [55]. Feedback regarding content depth and update frequency further indicates that users expect information to be directly relevant to their health status and screening decisions, as well as updated promptly to reflect the latest evidence and guidelines. Overall, these improvement suggestions emphasize the importance of usability, interactivity, and content relevance for high-risk populations for breast cancer, pointing to future optimization strategies such as enhancing interactive features, reorganizing information architecture for rapid access, and increasing content update frequency, thereby strengthening user engagement and the overall value of the app.

### Limitations

This study has several limitations. First, the small sample size from one hospital inherent in this pilot study limits the ability to draw definitive conclusions, and the preliminary effectiveness findings should therefore be interpreted as hypothesis-generating rather than confirmatory. Second, the enrolled high-risk populations for breast cancer in this study did not encompass individuals with genetic susceptibility or a history of chest radiotherapy. Subsequent studies should incorporate these groups to improve the generalizability of the results. Third, willingness to participate in screening was selected as the primary outcome, which represents behavioral intention rather than actual screening uptake. Future multicenter randomized controlled trials with longer follow-up periods are warranted to further evaluate the preliminary findings and to determine whether improvements in screening willingness translate into actual screening participation. Finally, the app was developed only for Android devices, which may limit the generalizability of feasibility and usability findings to iPhone users.

### Implications and Future Work

This pilot study demonstrates that the “Ruaikang” mobile health app exhibits good feasibility and usability among high-risk populations for breast cancer, providing preliminary signals of potential effectiveness in supporting health-related beliefs. The app is well-aligned with users’ core needs and may contribute to improvements in screening intention, although these effects should be interpreted cautiously, given the exploratory nature of the study. “Ruaikang” could serve as a key bridge connecting health care resources with high-risk populations for breast cancer. Through continuous refinement of health intervention strategies, it may facilitate broader adoption of early screening practices, alleviate the public health burden of breast cancer among Chinese women, and enhance their sense of health empowerment and overall well-being.

In future work, the research team will systematically refine the app’s core features, user interaction, content depth, and presentation based on feedback collected during the pilot phase. A larger-scale, multicenter randomized controlled trial with an expanded sample covering diverse geographic regions will be conducted to further validate these preliminary findings. In addition, a long-term follow-up mechanism will be established to monitor the sustainability of screening behaviors and changes in early detection rates, thereby generating longitudinal evidence to support the app’s continued development and broader implementation.

### Conclusions

This study demonstrated that the “Ruaikang” mobile health app is feasible and usable and provides preliminary signals of potential effectiveness for high-risk populations for breast cancer. The app may contribute to enhancing awareness and supporting willingness to participate in breast cancer screening among this population. Future research will optimize the app based on participant feedback and conduct studies with larger samples and longer follow-up periods to further examine the sustainability and potential impact on screening behaviors among high-risk populations for breast cancer.

## Supplementary material

10.2196/86429 Multimedia Appendix 1 The detailed information on the app’s development process, user side features, and back-end management functions.

10.2196/86429 Multimedia Appendix 2 Sensitivity analysis of generalized estimating equations models before and after multiple imputation.

10.2196/86429 Multimedia Appendix 3 Demographic and use characteristics of the participants in the qualitative study.

10.2196/86429 Multimedia Appendix 4 The results of the qualitative study.

10.2196/86429 Checklist 1 CONSORT checklist.

## Abbreviations

CONSORT: Consolidated Standards of Reporting Trials
GEE: generalized estimating equation
HBM: health belief model
NCCN: National Comprehensive Cancer Network

## References

[1] Bray F, Laversanne M, Sung H (2024). Global cancer statistics 2022: GLOBOCAN estimates of incidence and mortality worldwide for 36 cancers in 185 countries. CA Cancer J Clin.

[2] Wu Q, Fan BM, Li Y (2025). Analysis and interpretation of the 2022 Global Cancer Statistics Report: China and the world’s cancer disease burden and epidemiological trends. Theory Pract Diagn.

[3] Shen SJ, Sun Q, Huang X (2022). Chinese guidelines for breast cancer screening in women. J Chin Res Hosp.

[4] Pilewskie M, Eroglu I, Sevilimedu V, Le T, Mangino D, Morrow M (2024). Participation in a high-risk program is associated with a diagnosis of earlier-stage disease among women at increased risk for breast cancer development. Ann Surg Oncol.

[5] Cohen SY, Stoll CR, Anandarajah A, Doering M, Colditz GA (2023). Modifiable risk factors in women at high risk of breast cancer: a systematic review. Breast Cancer Res.

[6] Shi Q, Gao J, Yu H (2025). Characterization and functional analysis of BRCA1 and BRCA2 variants in a cohort of 100 unselected patients undergoing germline screening. Transl Oncol.

[7] Cardoso R, Hoffmeister M, Brenner H (2023). Breast cancer screening programmes and self-reported mammography use in European countries. Int J Cancer.

[8] (2019). Healthy China Initiative (2019–2030). National Health Commission of the People’s Republic of China.

[9] Kırca N, Tuzcu A, Gözüm S (2018). Breast cancer screening behaviors of first degree relatives of women receiving breast cancer treatment and the affecting factors. Eur J Breast Health.

[10] Zhang X, Yang L, Liu S (2021). Evaluation of different breast cancer screening strategies for high-risk women in Beijing, China: a real-world population-based study. Front Oncol.

[11] Zhao X, Meng HY, Sun GL (2019). Knowledge, attitude and practice on early screening for breast cancer of female first-degree relatives of breast cancer patients. Chin J Modern Nurs.

[12] Bevers TB, Niell BL, Baker JL (2023). NCCN Guidelines® insights: Breast Cancer Screening and Diagnosis, Version 1.2023. J Natl Compr Canc Netw.

[13] Noman S, Elarusy NME, Rahman HA (2024). Investigating the effect of the educational intervention based on the Health Belief Model on the knowledge and beliefs of Yemeni teachers in the use of breast cancer screening: a randomized controlled trial study. BMC Cancer.

[14] Kowitt SD, Ellis KR, Carlisle V (2019). Peer support opportunities across the cancer care continuum: a systematic scoping review of recent peer-reviewed literature. Support Care Cancer.

[15] Molokwu JC, Dwivedi A, Alomari A, Shokar N (2024). Effectiveness of a Breast Cancer Education Screening and NavigaTion (BEST) intervention among Hispanic women. Health Promot Pract.

[16] Hersch J, Barratt A, Jansen J (2015). Use of a decision aid including information on overdetection to support informed choice about breast cancer screening: a randomised controlled trial. Lancet.

[17] Baron RC, Rimer BK, Breslow RA (2008). Client-directed interventions to increase community demand for breast, cervical, and colorectal cancer screening a systematic review. Am J Prev Med.

[18] Aguiar-Ibáñez R, Mbous YPV, Sharma S, Chakali R, Chawla E (2025). Barriers to cancer screening uptake and approaches to overcome them: a systematic literature review. Front Oncol.

[19] Sprague BL, Ahern TP, Herschorn SD, Sowden M, Weaver DL, Wood ME (2021). Identifying key barriers to effective breast cancer control in rural settings. Prev Med.

[20] Tavakoli B, Feizi A, Zamani-Alavijeh F, Shahnazi H (2024). Factors influencing breast cancer screening practices among women worldwide: a systematic review of observational and qualitative studies. BMC Womens Health.

[21] Global strategy on digital health 2020-2025. World Health Organization.

[22] Baniasadi T, Hassaniazad M, Rostam Niakan Kalhori S, Shahi M, Ghazisaeedi M (2023). Developing a mobile health application for wound telemonitoring: a pilot study on abdominal surgeries post-discharge care. BMC Med Inform Decis Mak.

[23] Li B, Heydari K, Enichen EJ, Kvedar JC (2025). A mobile health application that supports a patient centered approach to cardiovascular risk management. NPJ Digit Med.

[24] Mugabirwe B, Flickinger T, Cox L, Ariho P, Dillingham R, Okello S (2021). Acceptability and feasibility of a mobile health application for blood pressure monitoring in rural Uganda. JAMIA Open.

[25] Yusuf A, P Iskandar YH, Ab Hadi IS, Nasution A, Lean Keng S (2022). Breast awareness mobile apps for health education and promotion for breast cancer. Front Public Health.

[26] Roh S, Lee YS, Kenyon DB (2023). Mobile web app intervention to promote breast cancer screening among American Indian women in the Northern Plains: feasibility and efficacy study. JMIR Form Res.

[27] Eldridge SM, Chan CL, Campbell MJ (2016). CONSORT 2010 statement: extension to randomised pilot and feasibility trials. Pilot Feasibility Stud.

[28] Julious SA (2005). Sample size of 12 per group rule of thumb for a pilot study. Pharm Stat.

[29] Malterud K, Siersma VD, Guassora AD (2016). Sample size in qualitative interview studies: guided by information power. Qual Health Res.

[30] Li XX, Du XW, Song W, Lu C, Hao WN (2020). Effect of continuous nursing care based on the IKAP theory on the quality of life of patients with chronic obstructive pulmonary disease. Medicine (Baltimore).

[31] Rosenstock IM, Strecher VJ, Becker MH (1988). Social learning theory and the Health Belief Model. Health Educ Q.

[32] Josefsson C, Liljeroos T, Hellgren M, Pöder U, Hedström M, Olsson EMG (2024). The Sukaribit smartphone app for better self-management of type 2 diabetes: randomized controlled feasibility study. JMIR Form Res.

[33] Wang Y, Lei T, Liu X (2020). Chinese System Usability Scale: translation, revision, psychological measurement. Int J Hum Comput Interact.

[34] Bolz J, Löscher A, Muhl R, Badke A, Predel HG, Perret C (2023). Feasibility, usability, and safety of ParaGym, an intelligent mobile exercise app for individuals with paraplegia: protocol for a pilot block-randomized controlled trial. JMIR Res Protoc.

[35] Chen Y, Xia HO, Oakley D (2007). A survey on women’s awareness of breast cancer and its early screening in Shanghai. Shanghai Nurs.

[36] Lu J, Ren H, Liu Y (2024). Knowledge, attitude, and willingness toward breast magnetic resonance imaging screening among women at high risk of breast cancer in Beijing, China. BMC Public Health.

[37] Ye X, Xu T, Cao J (2025). Knowledge, attitude, and practice toward genetic testing in breast cancer patients in China. PLoS One.

[38] Zhang X (2021). Study on Influencing Factors of Breast Cancer Screening Behavior Among Women Based on the Health Ecology Model [Master’s thesis]. https://tinyurl.com/2jdkmfza.

[39] Liao Y, Mohd Hairon S, Majdi Yaacob N, Alina Tengku Ismail T, Luo L (2025). Psychometric validation of a culturally adapted health belief model scale for breast cancer screening in Chinese women. PLoS One.

[40] Schwarzer R, Jerusalem M, Weinman J, Wright S (1995). Measures in Health Psychology: A User’s Portfolio Causal and Control Beliefs.

[41] Mystakidou K, Parpa E, Tsilika E, Galanos A, Vlahos L (2008). General perceived self-efficacy: validation analysis in Greek cancer patients. Support Care Cancer.

[42] Wang C (2001). General Self-Efficacy Scale: Chinese version and psychometric evaluation. Chin J Clin Psychol.

[43] Venkatesh V, Morris MG, Davis GB, Davis FD (2003). User acceptance of information technology: toward a unified view1. MIS Q.

[44] Anders C, Moorthy P, Svensson L (2024). Usability and user experience of an mHealth app for therapy support of patients with breast cancer: mixed methods study using eye tracking. JMIR Hum Factors.

[45] Willig C, Rogers WS (2017). The SAGE Handbook of Qualitative Research in Psychology.

[46] Hommel KA, Noser AE, Plevinsky J, Gamwell K, Denson LA (2024). Pilot and feasibility of the SMART IBD mobile app to improve self-management in pediatric inflammatory bowel disease. J Pediatr Gastroenterol Nutr.

[47] Salmani H, Ahmadi M, Shahrokhi N (2020). The impact of mobile health on cancer screening: a systematic review. Cancer Inform.

[48] High J, Grant K, Hope A (2024). Effects of an increased financial incentive on follow-up in an online, automated smoking cessation trial: a randomized controlled study within a trial. Nicotine Tob Res.

[49] Rezaee R, Asadi S, Yazdani A, Rezvani A, Kazeroon AM (2022). Development, usability and quality evaluation of the resilient mobile application for women with breast cancer. Health Sci Rep.

[50] Lustria MLA, Noar SM, Cortese J, Van Stee SK, Glueckauf RL, Lee J (2013). A meta-analysis of web-delivered tailored health behavior change interventions. J Health Commun.

[51] Galavi Z, Montazeri M, Khajouei R (2024). Which criteria are important in usability evaluation of mHealth applications: an umbrella review. BMC Med Inform Decis Mak.

[52] Galmarini E, Marciano L, Schulz PJ (2024). The effectiveness of visual-based interventions on health literacy in health care: a systematic review and meta-analysis. BMC Health Serv Res.

[53] Durmuş A (2024). The influence of digital literacy on mHealth app usability: the mediating role of patient expertise. Digit Health.

[54] Oakley-Girvan I, Yunis R, Fonda SJ (2023). Usability evaluation of mobile phone technologies for capturing cancer patient-reported outcomes and physical functions. Digit Health.

[55] Nuseibeh BZ, Johns SA, Shih PC, Lewis GF, Gowan TM, Jordan EJ (2024). Co-designing the MOSAIC mHealth app with breast cancer survivors: user-centered design approach. JMIR Form Res.

