# Supplementary material for: Feasibility, Usability, and Preliminary Effectiveness of an mHealth App to Promote Screening Behaviors Among High-Risk Populations for Breast Cancer: Randomized Controlled Pilot Study
Source: JMIR Mhealth Uhealth. 2026 Jul 14;14:e86429. doi: 10.2196/86429 (PMC13367949; doi:10.2196/86429)
Supplement: Multimedia Appendix 1 [file mhealth-v14-e86429-s001.docx]

**Development of the “Ruaikang” mobile health application**

There are three main development phases for the mobile health application, which have been outlined in Figure S1.

1. Preliminary integration of the resources included in the application. The research team employed an evidence-based nursing approach guided by the JBI Evidence-Based Healthcare Model [1] to systematically identify the high-quality evidence to promote screening engagement among high-risk populations for breast cancer. The evidence encompassed multiple aspects including knowledge education on breast cancer and screening, health beliefs promotion (such as enhancing perceived susceptibility, perceived severity, and self-efficacy), and fostering healthy behaviors. Meanwhile, a policy analysis was conducted to systematically gather and review policy documents related to breast cancer screening from official websites such as the National Cancer Center, the Chinese Anti-Cancer Association, and national and local Women’s Federations. Through this process, key supportive measures and policy themes in this field were identified. Additionally, health education materials on breast cancer were systematically integrated and refined based on authoritative guidelines, clinical education manuals, and professional literature to ensure comprehensiveness and professional rigor. By synthesizing insights from evidence-based research, policy analysis, and curated health education content, the team developed a preliminary resource repository designed to promote early screening behaviors among high-risk populations for breast cancer.
2. Identification and visualization of application resources. The research team invited six experts in breast cancer medical care to form an interdisciplinary panel. This panel evaluated and refined the preliminarily developed resource repository through two rounds of expert deliberation, resulting in a finalized version. The experts demonstrated 100% engagement and an average authority coefficient of 0.91, indicating high enthusiasm and strong credibility [2]. Subsequently, the research team visualized the resource content through various formats including videos, audio recordings, science-popularization animations, and posters.
3. Development and preliminary improvement of the application. The research team integrated the resource library as a core information module into the “Ruaikang” mobile health application and collaborated with software engineers to develop the application, including an Android client and a Web-based back-end management system. The research team conducted internal testing of the initial application version, providing recommendations for optimizing the interface design, functional architecture, and technical execution of core features. Through multiple rounds of debugging with engineers, the final version of the “Ruaikang” mobile health application was completed.


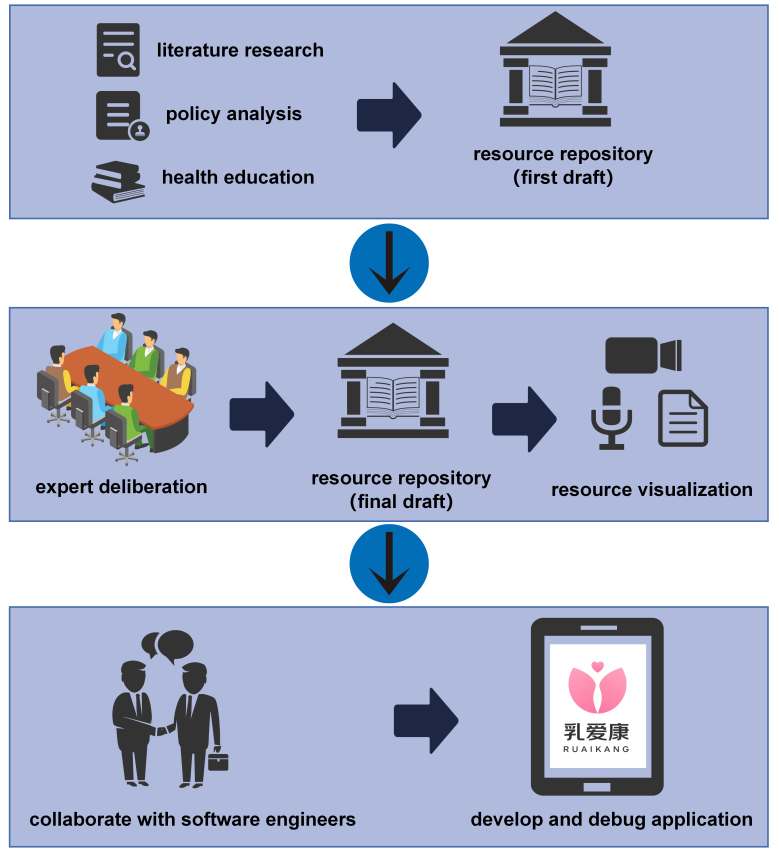


**Figure S1.** The development process of the “Ruaikang” mobile health application

**User side**

The user side of the application is designed to deliver breast health education, early screening guidance, health behavior promotion, and psychosocial support for high-risk populations for breast cancer. Featuring a modular interface, it comprises four core functional modules: the Resource Center, Health Center, Interaction Center, and Personal Center.

1. Resource Center. This module provides structured learning content covering breast anatomy, mechanisms of breast cancer development, early screening techniques and procedures, prevention strategies, relevant policies and regulations, as well as health belief improvement skills. Users can browse or search for resources on the “Discover” page, while the “Favorites” and “History” pages allow viewing saved items and past browsing activity respectively (Figure S2A). Each resource entry supports liking, bookmarking, and commenting. Links within the comment section also allow navigation to the Interaction Center (Figure S2B). After finishing a section, users may click the “Set goals” button to set personal health objectives and action plans independently (Figure S2C).


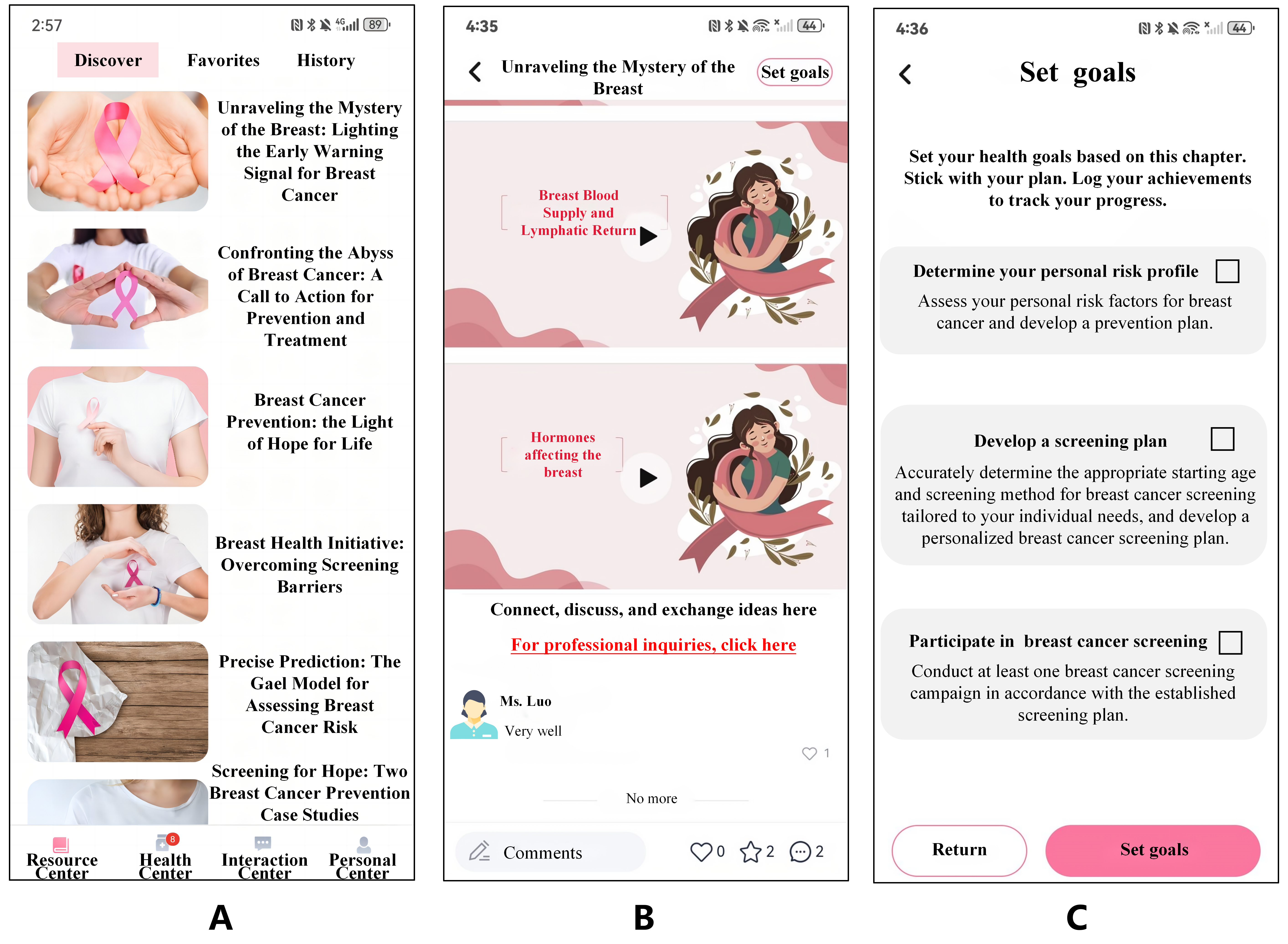


**Figure S2.** The interface of the Resource Center

1. Health Center. This module supports personalized health plan management by integrating user-defined goals and action plans. It allows users to track progress, revisit plans, and record check-ins for preset programs. Users can view plan execution status at any time and adjust their health management strategies accordingly. The interface of the Health Center was shown in Figure S3.


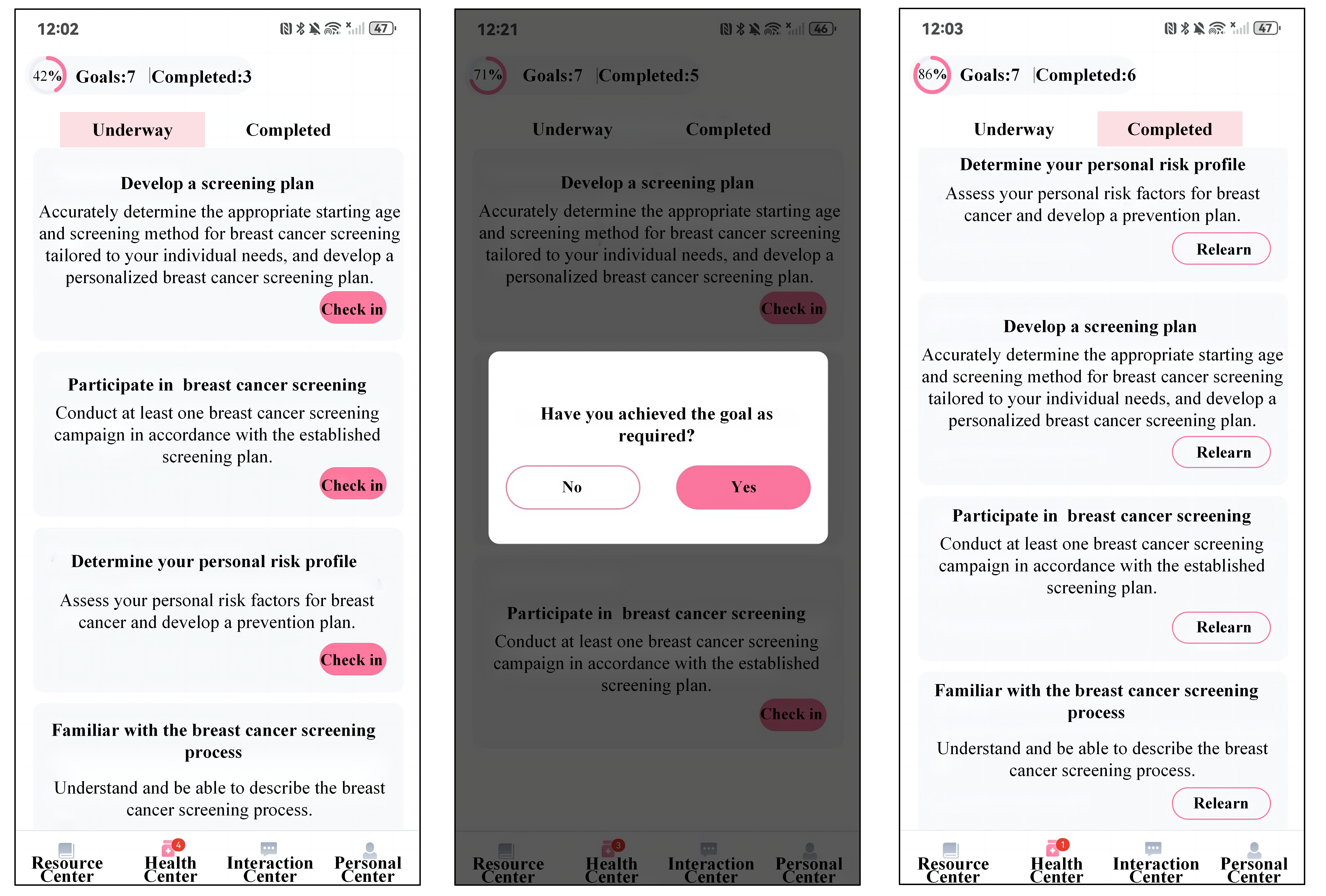


**Figure S3.** The interface of the Health Center

1. Interaction Center. This module provides an online communication platform between patients and healthcare professionals. It enables users to consult with certified medical teams regarding their personal health concerns and receive evidence-based professional guidance. The interface of the Interaction Center was shown in Figure S4A.
2. Personal Center. This module consolidates and displays the user’s personal information, resource browsing, collection, like, and comment history, along with a learning progress dashboard, forming a complete personal health management file. It also provides positive feedback on the health plans completed by users (such as displaying the encouraging slogan “You’re great!”). Users can also modify their personal information through this module. The interface of the Personal Center was shown in Figure S4B and S4C.


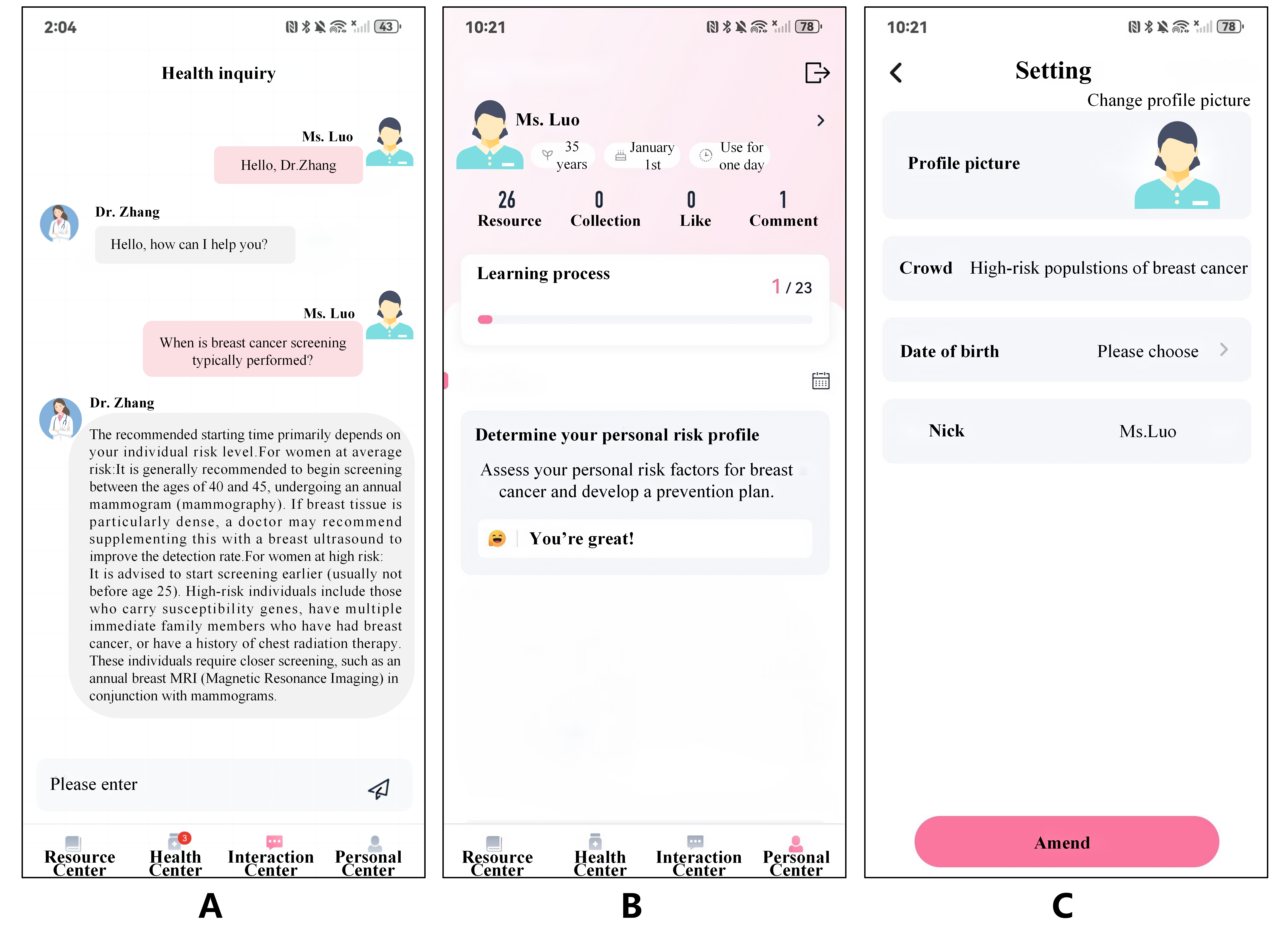


**Figure S4.** The interface of the Interaction Center and Personal Center

**Back-end management system**

The back-end management system is designed to oversee and control the overall operation of the application. The interface was shown in Figure S5. Its core functions include: (1) User Management. Monitor registered user information in real time, tracking key metrics including total registrations, login frequency, and resource access behavior. (2) Resource Management. Provide a resource publishing interface that supports dynamic updates and version control for breast health knowledge bases, psychosocial support resources, and behavioral intervention goals. (3) Health Consultation Management. Establish a professional consultation system to ensure that certified medical staff in the background can respond efficiently to users’ inquiries; and implements a comment review mechanism to ensure the accuracy and rigor of medical information dissemination on the platform. (4) System Configuration. Provide a global configuration function to manage key parameters such as software operation mode (development or production mode), user session timeout, resource effective learning time threshold, and time ranges for registration and login statistics.


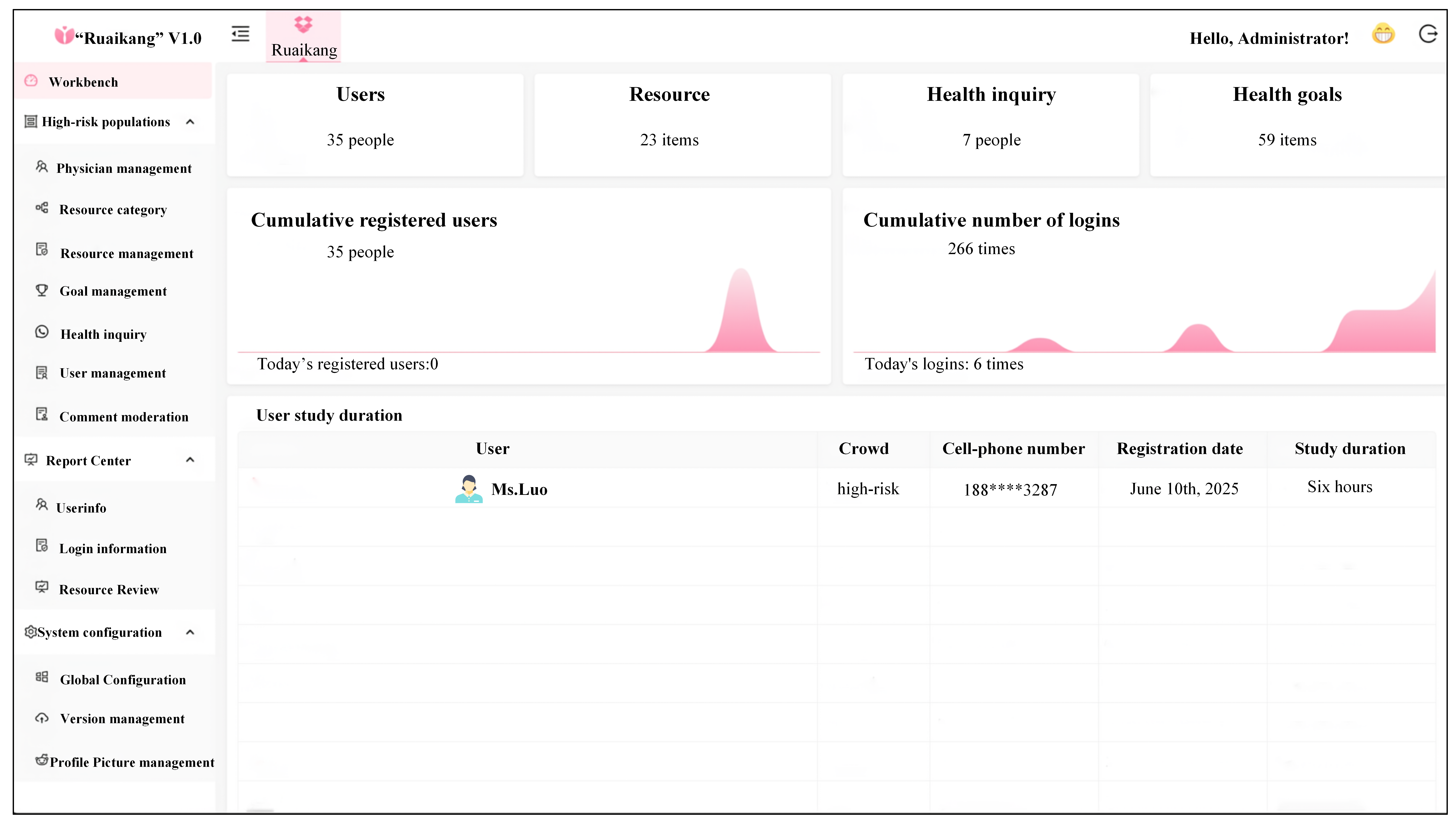


**Figure S5.** The interface of the back-end management system

**References**

1. Pearson A, Wiechula R, Court A, et al. The JBI model of evidence-based healthcare. Int J Evid Based Healthc. 2005;3(8):207-215. doi:10.1111/j.1479-6988.2005.00026.x
2. Zhou YL, Liu W, Li Y, et al. Progress and challenge of vaccine development against 2019-novel coronavirus (2019-nCoV). 2020;54(10):1081-1086. doi:10.3760/cma.j.cn112150-20200327-00453 (Chinese).
