# Supplementary material for: Feasibility, Usability, and Preliminary Effectiveness of an mHealth App to Promote Screening Behaviors Among High-Risk Populations for Breast Cancer: Randomized Controlled Pilot Study
Source: JMIR Mhealth Uhealth. 2026 Jul 14;14:e86429. doi: 10.2196/86429 (PMC13367949; doi:10.2196/86429)
Supplement: Multimedia Appendix 2 [file mhealth-v14-e86429-s002.docx]

**Sensitivity analysis of generalized estimating equations models before and after multiple imputation**

| Variables | Generalized estimating equation | | | | | Conclusion consistency^a^ |
| --- | --- | --- | --- | --- | --- | --- |
|  | Parameter | Multiple Imputation | | Original Data | |  |
|  |  | OR (95% CI)/β (95% CI) | *P* value | OR (95% CI)/β (95% CI) | *P* value |  |
| Willingness to participate in breast cancer screening | Group | 1.000 (0.330 - 3.033) | > .999 | 0.486 (0.139 - 1.704) | .26 | Consistent |
|  | T1 | 1.625 (0.966 - 2.735) | .07 | 1.000 (0.602 - 1.662) | > .999 |  |
|  | T2 | 1.625 (0.966 - 2.735) | .07 | 0.833 (0.587 - 1.182) | .32 |  |
|  | Group*T1 | 4.889 (1.343 - 17.801) | .02 | 10.971 (2.921 - 41.211) | < .001 |  |
|  | Group*T2 | 2.667 (0.717 - 9.912) | .14 | 9.143 (2.314 - 36.119) | < .01 |  |
| Breast cancer screening knowledge | Group | -0.360 (-1.816 - 1.096) | .63 | -0.275 (-1.831 - 1.281) | .73 | Consistent |
|  | T1 | 0.773 (0.055 - 1.491) | .03 | 0.636 (0.340 - 0.933) | < .001 |  |
|  | T2 | 1.268 (0.168 - 2.368) | .02 | 0.773 (-0.003 - 1.549) | .05 |  |
|  | Group*T1 | 7.557 (6.031 - 9.083) | < .001 | 6.732 (5.535 - 7.929) | < .001 |  |
|  | Group*T2 | 5.813 (4.055 - 7.570) | < .001 | 6.754 (5.305 - 8.203) | < .001 |  |
| Perceived susceptibility | Group | -0.520 (-1.058 - 0.018) | .06 | -0.426 (-1.061 - 0.209) | .19 | Consistent |
|  | T1 | 0.022 (-0.219 - 0.264) | .86 | 0.182 (0.021 - 0.343) | .03 |  |
|  | T2 | 0.238 (-0.011 - 0.487) | .06 | 0.273 (0.015 - 0.530) | .04 |  |
|  | Group*T1 | 1.835 (1.272 - 2.398) | < .001 | 1.766 (1.102 - 2.429) | < .001 |  |
|  | Group*T2 | 1.440 (0.848 - 2.032) | < .001 | 1.727 (1.057 - 2.398) | < .001 |  |
| Perceived severity | Group | -0.720 (-1.650 - 0.210) | .13 | -0.976 (-1.929 - -0.023) | .05 | Consistent |
|  | T1 | 0.396 (-0.165 - 0.957) | .17 | 0.773 (0.356 - 1.189) | < .001 |  |
|  | T2 | 0.995 (0.289 - 1.700) | .01 | 1.091 (0.413 - 1.768) | < .01 |  |
|  | Group*T1 | 2.634 (1.721 - 3.547) | < .001 | 3.017 (2.291 - 3.742) | < .001 |  |
|  | Group*T2 | 1.891 (0.713 - 3.070) | < .01 | 2.804 (1.899 - 3.709) | < .001 |  |
| Perceived benefits | Group | 0.320 (-0.502 - 1.142) | .45 | 0.371 (-0.539 - 1.280) | .42 | Consistent |

continued table

| Variables | Generalized estimating equation | | | | | Conclusion consistency^a^ |
| --- | --- | --- | --- | --- | --- | --- |
|  | Parameter | Multiple Imputation | | Original Data | |  |
|  |  | OR (95% CI)/β (95% CI) | *P* value | OR (95% CI)/β (95% CI) | *P* value |  |
|  | T1 | 0.479 (0.061 - 0.896) | .03 | 0.409 (-0.002 - 0.820) | .05 |  |
|  | T2 | 0.576 (0.017 - 1.135) | .04 | 0.545 (0.015 - 1.076) | .04 |  |
|  | Group*T1 | 1.236 (0.581 - 1.891) | < .001 | 1.380 (0.703 - 2.057) | < .001 |  |
|  | Group*T2 | 0.840 (0.055 - 1.626) | .04 | 0.981 (0.703 - 2.057) | .02 |  |
| Perceived barriers | Group | 1.120 (-0.182 - 2.422) | .09 | 1.469 (0.012 - 2.926) | .05 | Consistent |
|  | T1 | 0.007 (-0.447 - 0.462) | .98 | -0.273 (-0.459 - -0.087) | < .05 |  |
|  | T2 | -0.320 (-1.067 - 0.427) | .40 | -0.636 (-1.298 - 0.025) | .06 |  |
|  | Group*T1 | -3.351 (-4.502 - -2.199) | < .001 | -3.727 (-4.755 - -2.700) | < .001 |  |
|  | Group*T2 | -2.670 (-4.007 - -1.262) | <.001 | -3.258 (-4.500 - -2.017) | <.001 |  |
| Self-efficacy | Group | -0.880 (-3.769 - 2.009) | .55 | -0.055 (-3.363 - 3.253) | .97 | Consistent |
|  | T1 | 0.291 (-0.528 - 0.949) | .58 | 0.318 (0.026 - 0.611) | .03 |  |
|  | T2 | 0.647 (-1.006 - 2.299) | .44 | 0.273 (-0.593 - 1.138) | .54 |  |
|  | Group*T1 | 11.224 (8.952 - 13.496) | < .001 | 9.313 (7.592 - 11.035) | < .001 |  |
|  | Group*T2 | 8.206 (5.598 - 10.813) | < .001 | 9.306 (7.125 - 11.487) | < .001 |  |

^a^ “Consistent” indicates that the direction of the results remained unchanged before and after multiple imputation, although minor numerical differences existed between analyses. Therefore, the overall study conclusions were not affected by missing data. Note: OR: odds ratio, CI: confidence interval, β: regression coefficient, T0: baseline, T1: the fourth week, T2: the eighth week.
