# Supplementary material for: Feasibility, Usability, and Preliminary Effectiveness of an mHealth App to Promote Screening Behaviors Among High-Risk Populations for Breast Cancer: Randomized Controlled Pilot Study
Source: JMIR Mhealth Uhealth. 2026 Jul 14;14:e86429. doi: 10.2196/86429 (PMC13367949; doi:10.2196/86429)
Supplement: Multimedia Appendix 3 [file mhealth-v14-e86429-s003.docx]

**Demographic and usage characteristics of the participants in qualitative study (n = 8)**

| Participants | Age (years) | Educational attainment | Employment Status | History of breast cancer screening | Willingness to participate in breast cancer screening | Usage duration (hours) | Interview time (minutes) |
| --- | --- | --- | --- | --- | --- | --- | --- |
| S1 | 46 | High school/Junior college | Employed | Yes | Yes | 3 | 22 |
| S2 | 39 | Bachelor’s degree and above | Employed | No | Yes | 3 | 27 |
| S3 | 42 | Junior high school and below | Employed | No | Yes | 1 | 23 |
| S4 | 35 | High school/Junior college | Employed | No | Yes | 2 | 21 |
| S5 | 45 | High school/Junior college | Employed | No | No | 2 | 24 |
| S6 | 57 | Junior high school and below | Retired | Yes | Yes | 4 | 27 |
| S7 | 52 | Bachelor’s degree and above | Employed | No | Yes | 1 | 21 |
| S8 | 47 | High school/Junior college | Employed | No | No | 2 | 25 |

Note: S1 to S8 refer to the participants in the qualitative study.
