# Supplementary material for: Feasibility, Usability, and Preliminary Effectiveness of an mHealth App to Promote Screening Behaviors Among High-Risk Populations for Breast Cancer: Randomized Controlled Pilot Study
Source: JMIR Mhealth Uhealth. 2026 Jul 14;14:e86429. doi: 10.2196/86429 (PMC13367949; doi:10.2196/86429)
Supplement: Multimedia Appendix 4 [file mhealth-v14-e86429-s004.docx]

**The results of the qualitative study**

| Core themes | Sub themes | Code | Significant statement |
| --- | --- | --- | --- |
| Perceived value and acceptability of the application | Hybrid approach to promote effectiveness and long-term/sustained usage. | Content is professionally rigorous and highly practical | “Plain text alone can be hard to follow. With images, it’s much clearer. The content is comprehensive and well-structured, and really relevant to my needs, very practical.” (S1)  “The detailed self-examination video facilitates effective self-screening at home, promoting earlier detection of potential issues.” (S4)  “The Gail model assessment tool proves efficient: by completing the questionnaire it provides, I can quickly and accurately identify whether I fall into the high-risk category for breast cancer.” (S5)  “The breast cancer prevention tips provided are highly actionable. For instance, recommendations like avoiding staying up late, maintaining a healthy weight, and moderately increasing soy intake are all practical and manageable lifestyle changes I can adopt.” (S7) |
|  |  | Well-organized and comprehensive information | “The application provides comprehensive content, from disease awareness and prevention strategies to daily self-management strategies, eliminating the need to consult disparate online sources. This integrated approach offers considerable convenience.” (S4)  “The breast health educational content is comprehensive and well-organized, incorporating video explanations, case studies, and live demonstrations of self-examination steps, which are clearly outlined.” (S6) |
|  |  | The application is intuitive and user-friendly | “Furthermore, the user experience is very comfortable, with no unnecessary clutter, making all features readily accessible.” (S4).  “The operation is highly intuitive. Its user-friendly design ensures that even individuals with limited smartphone proficiency can use it effectively without a steep learning curve.” (S8) |

continued table

| Core themes | Sub themes | Code | Significant statement |
| --- | --- | --- | --- |
|  |  | Willingness to continue using the application | “People are paying more attention to self-care these days, and knowing about breast health is important for protection. The application provides professional, readily accessible information. I plan to use it often if it stays updated.” (S2)  “Breast health requires long-term attention from women. Given that the professional breast health content within this application is valuable, I am certain I will continue using it.” (S8) |
|  |  | Willingness to recommend the application actively | “If this breast health application continues to be helpful for me, I will also share it with others, after all, breast health is an issue of great importance.” (S1)  “It is comprehensive and practical, useful for helping my mother and friends gain knowledge and reduce anxiety. I’ll definitely recommend it.” (S2)  “I’m especially eager to recommend it. Once the application is fully optimized, it will undoubtedly become more and more useful. So I’ll keep using it going forward and will actively recommend it to others.” (S5)  “I’d definitely recommend it! The content on the application is all practical and useful, no cluttered ads. It helps me avoid unnecessary detours and better manage my breast health.” (S8) |
|  | Significant health promotion effectiveness | Raise awareness of breast health | “It encourages me to be more mindful of my breast health and to consider a mammogram in the future.” (S1)  “Thanks to the self-examination guide, I now check myself every week. It makes me feel much more secure.” (S5) |

continued table

| Core themes | Sub themes | Code | Significant statement |
| --- | --- | --- | --- |
|  |  | Increase breast cancer knowledge | “Prior to this, I had little to no knowledge of breast health. After engaging with the health application, I addressed and clarified my misconceptions regarding the causes and types of breast cancer, thereby gaining an objective understanding of the disease.” (S3)  “The application taught me the warning signs for breast cancer and how to prevent it. It clearly explained the screening process, including when and how to get checked, which was so valuable.” (S5) |
|  |  | Promote the development of healthy habits | “ I have become more attentive to specific aspects of breast health in my daily life than I was previously.” (S2)  “Now I make more mindful, healthier choices, like buying more of the recommended vegetables when shopping for groceries, controlling my dinner portions, and sticking to my daily health tracking. It’s all helping me build solid habits.” (S8) |
| System limitations and user-centered improvement needs | Lack of active engagement and interactions | Add AI-powered Q&A functionality | “I would like to see AI-powered features added, such as an instant Q&A system or a chatbot.” (S3) |
|  |  | Optimize the incentive mechanism | “Additionally, incorporating incentives into the Health Center, for example, rewarding users with a free consultation upon completing a health plan, would further boost engagement and support self-management.” (S3) |
|  |  | Lack of a platform for user-to-user communication | “The interactive center lacks a section for users to discuss issues, and users cannot view others’ shared experiences or respond to questions. I hope to see an interactive space where people can talk about problems they encounter and learn how others have solved them.” (S6) |
|  | Functional and content-related limitations | Health Center Content is hollow | “The Health Center in particular feels empty when I click into it, and using it is unfulfilling. I hope more content can be added.” (S6) |

continued table

| Core themes | Sub themes | Code | Significant statement |
| --- | --- | --- | --- |
|  |  | The interface is cluttered, and the sections are not clearly defined | “The interface presents a cluttered layout and poor submodule separation.” (S6) |
|  |  | Add video playback speed adjustment feature | “Also, the lack of a video speed control function is very inconvenient for those of us who prefer watching content at a faster pace.” (S6) |
|  |  | Lack a direct access to common content | “Sometimes I can’t find the information I need, and I don’t even know where to look.” (S1)  “The dietary recommendations are buried within a broad category and lack a direct access point. Creating a more visible shortcut for such frequently used content would save significant time.” (S5) |
|  |  | The design lacks originality | “Furthermore, the identical, unchanging logo on every resource cover is highly repetitive. This lack of variation leads to visual fatigue over time.” (S6) |
|  |  | Provide diverse content summaries to enhance information retrieval efficiency | “It would be helpful to summarize the key points from the video into a separate format, like a flowchart or a slide deck, to distill the core content for quick comprehension.” (S5)  “The video lacks accompanying PowerPoint slides, forcing me to patiently scan through the entire content. This made it very time-consuming to locate specific information, resulting in extremely low efficiency.” (S6)  “When it’s inconvenient to watch videos, it would be helpful to include text summaries alongside each video. This way, I can clearly understand the content just by reading the text and looking at the images.” (S8) |
|  |  | Specialized content such as breast cancer staging and symptoms requires refinement | “Regarding breast cancer staging, I hope to see clearer explanations for each stage’s definition, symptoms, and management approaches. The current symptom illustrations are only in cartoon form, I recommend adding realistic image options to make the content more comprehensive and meet diverse needs.” (S4) |

continued table

| Core themes | Sub themes | Code | Significant statement |
| --- | --- | --- | --- |
|  |  | Refine the content of policies related to breast health | “In the policy support section, I hope the core knowledge points can be further refined, such as listing the required content in bullet points.” (S5)  “The application would be more practical if it provided more granular detail on policies specific to different cities.” S7) |
|  |  | Update content promptly | “Furthermore, during my use of the application, I haven’t observed any updates. I hope to see more of the latest research findings included.” (S7) |
